# Black hairy tongue treated with traditional Chinese medicine: A case report

**DOI:** 10.1097/MD.0000000000034430

**Published:** 2023-10-27

**Authors:** Po-Yuan Kuo, Ping-Ho Chen, Shan-Hong Wu

**Affiliations:** a Department of Traditional Chinese Medicine, Taipei Medical University Hospital, Taipei, Taiwan

**Keywords:** black hairy tongue, case report, Chinese herbal medicine, traditional Chinese medicine

## Abstract

**Rationale:**

Black hairy tongue (BHT) is a rare condition that is conventionally managed by discontinuing associated medications or habits and practicing good oral hygiene, including tongue brushing. Previous studies have indicated that black tongue coating is often associated with gastrointestinal discomfort, which traditional Chinese medicine (TCM) could be a potentially effective option for treating this condition with minimal side effects. We present a case of BHT diagnosed and treated within 2 weeks by using TCM methods.

**Patient concerns:**

A 73-year-old woman presented with a chief concern of a black tongue that had persisted for 2 weeks and was accompanied by thirst and diarrhea. These symptoms were initially observed during her hospitalization for the treatment of staghorn calculi, xanthogranulomatous pyelonephritis, and urosepsis. Using the “four diagnostic methods” of TCM, we observed that her tongue had a thick black coating surrounded by a thick white coating; her tongue was of medium size with tooth marks, and its body color was light red.

**Diagnoses, interventions, and outcomes:**

On the basis of TCM theory, we made a clinical diagnosis of BHT and “spleen Qi deficiency with turbid dampness,” which may have been caused by the antibiotic treatment during hospitalization. Subsequently, we administered a Chinese herbal medicine (CHM) formula comprising a combination of Wu-Ling-San (五苓散) and a modification of Da-Yuan-Yin (達原飲). After 2 weeks of CHM treatment, the patient’s BHT was nearly eliminated, and the thick white coating and the corresponding symptoms were ameliorated. After 4 weeks of CHM treatment, the BHT was completely resolved.

**Lesson and conclusion:**

We present a case of BHT, a benign condition that may be caused by antibiotics. The literature does not contain reports on TCM-based diagnosis and treatment strategies for BHT. Using the 4 diagnostic methods of TCM, we observed that BHT was associated with gastrointestinal symptoms, which is consistent with the TCM theory. Moreover, CHM treatment rapidly relieved BHT and related symptoms without adverse events.

## 1. Introduction

Black hairy tongue (BHT) is a benign condition that is characterized by a dark and hairy growth on the lingual dorsum; specifically, it involves the hypertrophy and elongation of filiform papillae. This condition was first described by Amatus Lusitanus in 1557.^[1]^ The description of black tongue was also found in the first surviving tongue monograph Ao Shi Shang Han Jin Jing Lu (敖氏傷寒金鏡錄), which was edited and completed in 1341.^[2]^ The reported prevalence of BHT ranges from 0.6% to 11.3% in different populations.^[3,4]^ BHT may be induced by lifestyle habits (e.g., practicing poor oral hygiene; being on a diet of soft or pureed food; smoking; or consuming black tea, coffee, or alcohol), medical conditions (e.g., immunocompromised states, systemic infection, trigeminal neuralgia, or dry mouth), or medication use (e.g., antibiotic or antixerostomia agents).^[4]^ First-line management strategies for BHT include discontinuing BHT-inducing drugs, gentle brushing the tongue, and practicing good oral hygiene.^[4,5]^ Second-line management strategies involve therapeutic interventions, including the administration of topical (e.g., triamcinolone acetonide or salicylic acid) or oral (e.g., retinoids, antifungals, or antibiotics) agents,^[4]^ photo-therapy,^[6]^ mechanical removal therapy (e.g., dental water jet,^[7]^ electrocautery dissection^[8]^).

It was reported that BHT was associated with some gastrointestinal symptoms such as anorexia, nausea,^[9]^ halitosis,^[10]^ xerostomia and tingling sensation,^[11]^ gagging, tickling sensation, dysgeusia.^[4,12]^ In addition, a cross-sectional Chinese study of 179 cases indicated a correlation between black hairy tongue and gastrointestinal disorders,^[13]^ and many case reports have also reported that black hairy tongue is accompanied by gastrointestinal symptoms.^[9–11]^ Traditional Chinese medicine (TCM) has been shown to have good efficacy in treating gastrointestinal diseases such as dyspepsia^[14]^ and dry mouth,^[15]^ with minimal side effects. Therefore, it may be worth considering using TCM in the treatment of black hairy tongue.

In TCM, the “four diagnostic methods”—comprising inspection, auscultation and olfaction, inquiry, and pulse-taking and palpation—are typically used for holistic patient assessment. BHT diagnosis in TCM not only entails assessing changes in a patient’s tongue but also involves conducting a full-body assessment to identify relevant “TCM patterns” and then administer the appropriate treatment. Moreover, TCM includes a unique tongue diagnosis technique that entails analyzing the shape, color, coating, and moisture of the tongue to infer physiological or pathological changes in the body.^[16]^ The tongue coating is predominately composed of filiform papillae, desquamated epithelial cells, and food particles and can vary extensively owing to factors such as oral microorganisms, blood metabolites, and salivary secretions.^[17]^ Tongue coating is also related to intestinal microbiota and multiple systemic diseases.^[18]^ In TCM theory, the tongue coating is considered to be produced by “Stomach Qi,” which is related to gastrointestinal function; hence, the tongue coating is typically employed as an indicator for monitoring gastrointestinal function.^[19]^

The literature does not contain a comprehensive report on TCM-based diagnosis and treatment outcomes for BHT. Accordingly, to fill this gap, this paper presents a case of BHT with gastrointestinal symptoms treated with Chinese herbal medicine (CHM).

## 2. Patient Information

A 73-year-old woman presented to the outpatient TCM clinic of a university hospital in Taipei on April 14, 2021. At her presentation, the patient had a chief concern of a black tongue that had lingered for 2 weeks, in addition to reporting that she experienced thirst and diarrhea. She had a history of hypertension and arrhythmia that were under regular medical control; several years prior to her presentation, she had received an appendectomy and uterine myomectomy without sequelae. A psychosocial history assessment did not reveal any notable abnormality in the patient’s family. Moreover, she had no history of alcohol consumption, and she was a nonsmoker.

On March 14, 2021 (which was 1 month prior to her presentation to our TCM clinic), the patient presented to our emergency department with a fever, chills, and dysuria. Examination results revealed a fever of up to 38.6°C, heart rate of 124/min, respiratory rate of 23/min, and blood pressure of 215/88 mm Hg. Furthermore, laboratory test results revealed leukocytosis (white blood cell count: 17,360; neutrophil count: 91.9%), anemia (hemoglobin level: 9.2 g/dL), and mildly increased creatinine levels (17.0 mg/dL) and blood urea nitrogen levels (1.0 mg/dL). The patient’s liver parameters were within the normal range. A computed tomography scan of the urinary system indicated the presence of right staghorn calculi with hydronephrosis and perirenal abscess extension, which are associated with xanthogranulomatous pyelonephritis. Accordingly, the patient was diagnosed as having right staghorn calculi, xanthogranulomatous pyelonephritis, and urosepsis.

The patient was subsequently administered an antibiotic (ceftriaxone, 2 g; administered intravenous daily) and pain relievers. Moreover, a pigtail catheter was inserted under computed tomography guidance for perirenal abscess drainage. The patient was then admitted to our intensive care unit for further medical care.

During her intensive care unit admission, the patient experienced intermittent fever and right flank pain. The daily drainage volume from the pigtail catheter was 50 to 100 mL. On day 3 after admission, ceftriaxone was switched to flumarin (1 g; administered intravenous every 8 hours). The patient also experienced constipation and abdominal bloating, which were treated with a stool softener.

On day 10 after admission, the patient’s fever subsided, and when her condition stabilized, she was transferred to a normal ward. A successful right nephrectomy was performed on day 17 after admission. However, the patient noted that the surface of her tongue had gradually become black. The patient was discharged on day 26 with a 2-week course of antibiotics (cefixmycin, 100 mg; 2 tablets consumed orally twice a day), pain reliever, and stool softener. Follow-up visits to the urology clinic revealed that her BHT was not resolved; accordingly, she was admitted to our TCM clinic under the urologist’s suggestion.

## 3. Clinical findings

**Figure 1.**
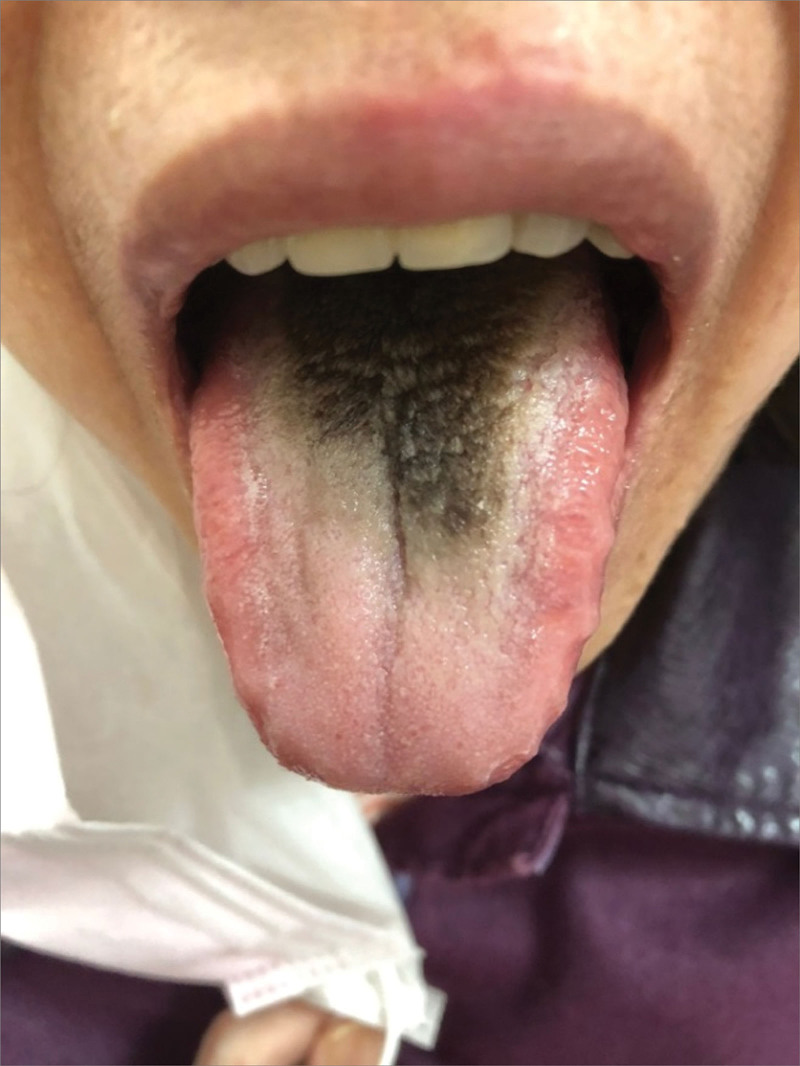
Black hairy tongue during the first visit. The tongue had a thick black coating surrounded by a thick white coating with tooth marks.

The patient denied a history of oral disease or poor oral hygiene. Additionally, she neither smoked nor consumed dark-colored food, beverages, or substances such as coffee, tea, chocolate, or TCM agents. Upon inspection, we noted black elongated hair-like filiform papillae on the lingual dorsum (Fig. 1).

We used the 4 diagnostic methods (i.e., inspection, auscultation and olfaction, inquiry, and pulse-taking and palpation) of TCM to assess the patient’s tongue for diagnosis. The inspection method revealed a thick black coating (furry) surrounded by a thick white coating on the patient’s tongue; moreover, her tongue was of medium size with tooth marks, and its body color was light red. The auscultation and olfaction method revealed no obvious abnormalities. Moreover, the inquiry method revealed similar results to those of the aforementioned tests. Finally, the pulse-taking method revealed that the patient’s pulse was “slippery” and had a rapid rate.

## 4. Timeline

We have compiled the patient’s medical history into a table to present it in chronological order (Table 1).

**Table 1:** The timeline of the patient’s clinical event and the development and management of BHT.

Before CHM treatment			
Date	Clinical event		Intervention
March 14, 2021	The patient visited the emergency department and was diagnosed as having right staghorn calculi, xanthogranulomatous pyelonephritis, and urosepsis		Ceftriaxone (2 g, administered intravenously daily), pain reliever, and pigtail catheter drainage
March 16, 2021	After ICU admission, the patient experienced intermittent fever, in addition to experiencing constipation and abdominal bloating		Flumarin (2 g, administered intravenously every 8 h) as a replacement for the antibiotics, pain reliever, and stool softener
March 23, 2021	The fever subsided, and the patient was transferred to a normal ward because her condition was stable		Flumarin (2 g, administered intravenously every 8 h), pain reliever, and stool softener
March 30, 2021	The patient underwent surgery conducted to treat the aforementioned health problems, and she developed BHT after the surgery		Right nephrectomy, flumarin (2 g administered intravenously every 8 h), pain reliever, and stool softener
April 8, 2021	The patient was discharged		Cefixmycin (100 mg, 2 tablets consumed orally twice a day), pain reliever, and stool softener for 2 wk
CHM treatment			
Date	Summaries from initial and follow-up visits	Diagnostic testing	Intervention
April 14, 2021	The patient visited the TCM clinic with a chief concern of a black tongue that had persisted for 2 wk and was accompanied by thirst and diarrhea	The 4 diagnostic methods of TCM were used: • An inspection of the tongue revealed a thick black coating surrounded by a thick white coating; the tongue was of medium size with tooth marks, and its body color was light red • Pulse-taking and palpation revealed a slippery and rapid pulse	CHM formula comprising a combination of WLS and a modified version of DYY
April 21, 2021	The patient’s thirst and diarrhea continued	The BHT area was reduced	CHM treatment for another 1 wk
April 28, 2021	The patient’s thirst and stool passage frequency were ameliorated	The patient’s BHT was nearly eliminated, and the thick white coating was reduced	CHM treatment for another 2 wk
May 12, 2021	The patient reported experiencing insomnia for several days Her thirst and stool passage frequency were ameliorated	The patient’s BHT was completely resolved. The tongue was redder than before, and the tongue coating became thicker than before. These observations corresponded to “excess heat”	*Artemisia annua* (青蒿) as a replacement of *Atractylodes lancea*
Around May 26, 2021	The CHM treatment was stopped		
September 8, 2021	The patient visited the clinic with concerns of dyspepsia and insomnia. The patient’s thirst and diarrhea were resolved. No further BHT recurrence was noted	A white coating with a thick center was noted	

BHT = black hairy tongue, CHM = Chinese herbal medicine, DYY = Da-Yuan-Yin, ICU = intensive care unit, TCM = traditional Chinese medicine, WLS = Wu-Ling-San.

## 5. Diagnostic assessment

The black hairy coating appeared on the lingual dorsum and was thus clinically diagnosed as BHT syndrome. After ruling out common etiologies such as poor oral hygiene, substance or food intake, and medical conditions, we speculated that the antibiotics used during her previous treatment most likely caused her BHT.

In tongue diagnosis in TCM, a light-red tongue body is considered to be normal. However, our patient’s tongue had a thick black coating surrounded by a thick white coating, indicating “turbid dampness”; her tongue was also of medium size with tooth marks, indicating “spleen Qi deficiency.” Accordingly, on the basis of TCM principles regarding tongue diagnosis, the patient was diagnosed as having “spleen Qi deficiency with turbid dampness.” In addition to BHT, the patient reported feeling thirsty (and drinking water could not relieve her thirst) and having diarrhea with stool passage several times a day. On the basis of these symptoms, she was diagnosed as having “spleen Qi deficiency,” which leads to abnormal water distribution in the body.

BHT is generally a benign condition. To identify a diagnostic marker of BHT in TCM, the basic appearance of the tongue must be assessed. As mentioned, in our patient, we observed that the black hairy coating was surrounded by a thick white coating, which signified that her BHT was induced by the aggravation of the white coating. Accordingly, we identified that BHT involves the presence of “turbid dampness.”

A study reported that antibiotics such as cephalosporin, tetracycline, clarithromycin, penicillin, erythromycin, doxycycline, linezolid, and neomycin cause BHT.^[4,5]^ Our patient was prescribed 3 different cephalosporins for infection treatment, which may have caused her BHT.

## 6. Therapeutic intervention

After diagnosing “spleen Qi deficiency with turbid dampness,” we administered a concentrated CHM formula in granular form to the patient. This formula comprised a combination of Wu-Ling-San (WLS, 五苓散) and a modification of Da-Yuan-Yin (DYY, 達原飲); it was administered at a dose of 4.8 g per pack 3 times a day orally (Table 2) for 1 week. The formula was covered by Taiwan’s National Health Insurance program and was provided by a pharmaceutical company that was certified as following Good Manufacturing Practice standards.

**Table 2:** Components of CHM prescription.

CHM formulas	Botanical name	Chinese name
WLS	Rhizoma Alismatis	澤瀉
	Wolfiporia hoelen	茯苓
	Polyporus umbellatus	豬苓
	Ramulus Cinnamomi cassiae	桂枝
	Rhizoma Atractylodis macrocephalae	白朮
Modified version of DYD	Anemarrhena asphodeloides	知母
	Scutellaria baicalensis	黃芩
	Lanxangia tsaoko	草果
	Areca catechu	檳榔
	Magnolia officinalis	厚朴
	Atractylodis rhizoma	蒼朮

DYY = Da-Yuan-Yin, WLS = Wu-Ling-San.

WLS and DYY have been commonly prescribed by TCM practitioners for hundreds of years. WLS has been extensively used to treat “spleen Qi deficiency and dampness.”^[20]^ A previous study reported that WLS can be used to treat renal problems such as edema, uremia, nephrosis, and uncomfortable urination and that it can be used to treat gastrointestinal problems such as thirst, vomiting, and diarrhea.^[21]^ DYD was developed for treating “turbid dampness” and has often been prescribed for treating respiratory infections such as COVID-19 infection, pneumonitis, and gastroenteritis.^[22]^ For our patient, we modified the original component of DYY to suit the patient’s condition.

**Figure 2.**
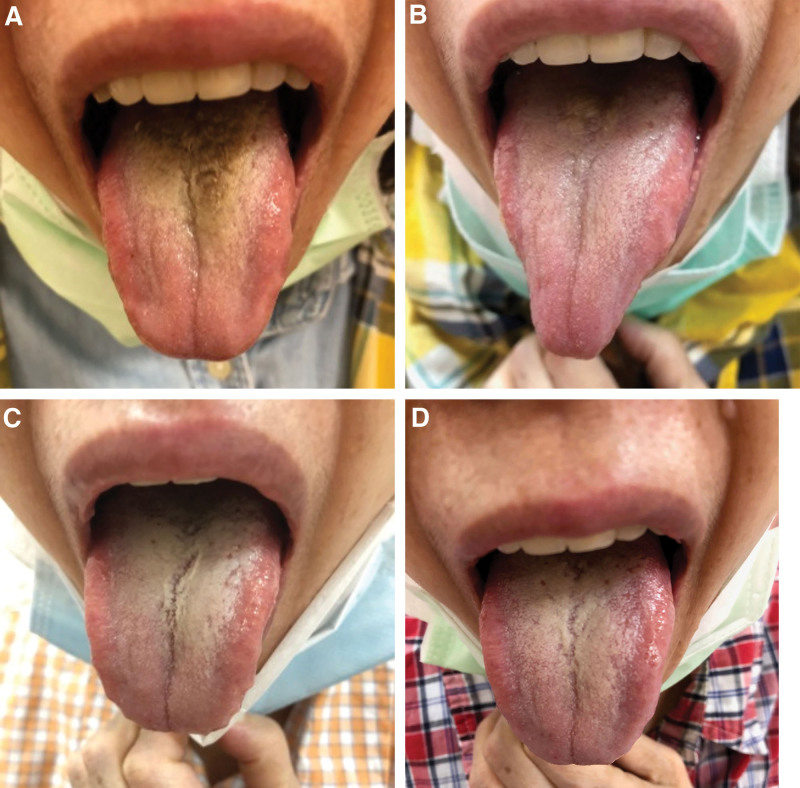
(A) After 1 week of CHM administration, the BHT area decreased. (B) After 2 weeks of CHM administration, the BHT area was nearly eliminated, and the thick white coating area was also reduced. (C) After 4 weeks of CHM administration, the BHT area and thick white coating were completely resolved, and the patient’s thirst and stool frequency also decreased. (D) Approximately 5 months after the first CHM administration, the patient returned for a follow-up visit 3 months after discontinuing the medication, but no recurrence of BHT was observed. BHT = Black hairy tongue, CHM = Chinese herbal medicine.

We observed that after 1 week of CHM administration, the BHT area decreased. Hence, CHM administration was continued for another week (Fig. 2A). After 2 weeks of CHM administration, the BHT area was nearly eliminated, and the area of the thick white coating was also reduced. In addition, the patient’s thirst and stool passage frequency were reduced (Fig. 2B). After 4 weeks of CHM administration, the BHT was completely resolved. However, after treatment, the patient experienced insomnia for several days; her tongue had become redder than it was before treatment, and the coating of her tongue became thicker. Accordingly, we replaced *Atractylodes lancea* with *Artemisia annua* (青蒿) to relieve “excess heat” symptoms (Fig. 2C). The treatment was discontinued on May 26, 2021. On September 8, 2021 (approximately 5 months after the first CHM administration), the patient visited our clinic with concerns about dyspepsia and insomnia. We observed that the patient’s thirst and diarrhea were resolved, and we did not note BHT recurrence (Fig. 2D).

## 7. Follow-up and outcomes

A follow-up assessment conducted 6 months after the first CHM treatment revealed that the patient’s chief concern was resolved without any recurrence. In addition, we performed the 4 diagnostic methods of TCM again, and the results did not reveal the previously identified “spleen Qi deficiency and turbid dampness.” The patient did not experience adverse events during the CHM treatment process and was satisfied with the treatment outcomes; she also demonstrated favorable adherence to the treatment.

## 8. Discussion

The 4 diagnostic methods of TCM can enable practitioners to perform a comprehensive assessment of a patient’s health status. In TCM, oral hygiene and imbalanced body constitution are considered to be associated with BHT. CHM treatment can effectively resolve BHT and its associated symptoms with few adverse events.

Antibiotics have various effects, including the reduction of microbial diversity and amelioration of inflammation, which can be considered to be related to the “Cold” pattern in TCM. In addition, both oral and intravenous antibiotics engender adverse drug reactions—including diarrhea, nausea, vomiting, and abdominal pain—in the gastrointestinal system,^[23]^ and these are considered to be associated with “stomach and spleen Qi” in TCM.

Although most patients with BHT are asymptomatic, studies have reported that BHT is associated with gastrointestinal symptoms such as gagging, nausea, tickling sensation, dry mouth, dysgeusia, and halitosis.^[4,12]^ A case series of 179 patients with BHT reported that 57.5% of the patients had digestive disorders.^[13]^ The correlation between BHT and digestive disorders is consistent with TCM theory that the coating of the tongue is produced by “stomach Qi.” In our case study, we noted that our patient experienced digestive disorders such as constipation, bloating, dry mouth, and diarrhea after being hospitalized; these disorders may have been induced by the administered antibiotics, which caused “spleen Qi deficiency.”

This study has some limitations. We observed that most of the symptoms of antibiotic-induced BHT could be resolved after the discontinuation of antibiotics, as reported by previous studies^.[3–5,11]^ However, the time required for recovery from BHT after the discontinuation of antibiotics was unclear and could vary from as little as 3 days to more than 4 weeks, despite the patient practicing good oral hygiene, including tongue brushing.^[5]^ Despite this uncertainty, we can confirm the effectiveness of CHM treatment in relieving BHT-associated symptoms (e.g. thick fur, thirst, and diarrhea) and ameliorating BHT.

## 9. Conclusion

We present a case of BHT—a benign condition—possibly induced by antibiotic use. According to our review of the literature, no study has reported TCM-based diagnosis and treatment of BHT. Using the 4 diagnostic methods of TCM, we observed that BHT was associated with gastrointestinal symptoms, which is consistent with TCM theory. CHM treatment rapidly relieved our patient’s BHT and associated symptoms without adverse events.

## Author contributions

**Conceptualization:** Ping-Ho Chen.

Project administration: Po-Yuan Kuo.

Supervision: Shan-Hong Wu.

Writing – original draft: Po-Yuan Kuo.

Writing – review & editing: Po-Yuan Kuo, Shan-Hong Wu.
